# Pulmonary Pseudosequestration in a Child with Down Syndrome

**DOI:** 10.3390/children12121667

**Published:** 2025-12-08

**Authors:** Virginia Mirra, Rosamaria Terracciano, Alessia Spagnoli, Pierluigi Vuilleumier, Fabio Antonelli, Elvira Calabrese, Margherita Rosa, Annalisa Allegorico

**Affiliations:** 1 Chronic and Multifactorial Diseases Unit, Santobono-Pausilipon Children’s Hospital, 80129 Naples, Italy; virginia.mirra@hotmail.it (V.M.); e.calabrese@santobonopausilipon.it (E.C.); m.rosa@santobonopausilipon.it (M.R.); 2 Department of Translational Medicine, Section of Pediatrics, University of Naples “Federico II”, 80131 Naples, Italy; rosamariaterracciano@libero.it; 3 Unit of Pediatric Pneumology and UTSIR, Santobono-Pausilipon Children’s Hospital, 80129 Naples, Italy; p.vuilleumier@santobonopausilipon.it (P.V.); fabantonelli65@gmail.com (F.A.); a.allegorico@santobonopausilipon.it (A.A.)

**Keywords:** Down syndrome, pulmonary sequestration, pseudosequestration, congenital lung malformation

## Abstract

**Highlights:**

**What are the main findings?**

- First documented association between Down syndrome and intralobar pulmonary pseudosequestration.
- CT angiography revealed an anomalous systemic arterial supply arising from the celiac trunk, with venous drainage into the pulmonary veins and partial communication with the bronchial tree.

**What are the implications of the main findings?**

- Suggests a possible shared developmental mechanism between trisomy 21-related vascular dysregulation and congenital pulmonary malformations.
- Underscores the importance of considering pulmonary sequestration in patients with Down syndrome who present with recurrent or unexplained respiratory distress.

**Abstract:**

Background: Down syndrome (DS) is commonly associated with complex respiratory phenotypes due to anatomical, immunological, and vascular factors. Pulmonary sequestration (PS) is a rare congenital malformation of non-functioning lung tissue with anomalous systemic arterial supply, occasionally reported in syndromic individuals. Case presentation: We report the case of a female infant with DS who developed acute respiratory distress secondary to respiratory syncytial virus infection. Chest imaging revealed an intralobar pulmonary pseudosequestration in the right lower lobe, supplied by the celiac trunk and draining into the pulmonary veins, with a communication to the bronchial tree. The patient required pediatric intensive care support and nutritional rehabilitation. Surgical resection was deferred until adequate weight optimization could be achieved. Discussion: This is, to our knowledge, the first description of intralobar pulmonary pseudosequestration in a patient with DS. The association suggests possible overlapping developmental mechanisms involving abnormal angiogenesis and emphasizes the importance of considering congenital pulmonary malformations in DS patients presenting with recurrent or severe respiratory symptoms. Conclusions: Early recognition and tailored management may improve clinical outcomes in this vulnerable population.

## 1. Introduction

Down syndrome (DS), caused by trisomy of chromosome 21, is the most common chromosomal disorder worldwide [1,2]. It is characterized by multisystem involvement including characteristic craniofacial features, hypotonia, cognitive impairment, and high prevalence of congenital malformations—particularly cardiac and gastrointestinal [1,2]. Congenital heart defects (CHDs) are observed in approximately 45–50% of newborns with DS, with atrioventricular septal defects being the most frequent. The prevalence of CHDs varies by ethnicity and sex [3,4,5].

Respiratory disease is the leading cause of morbidity and mortality across all age groups in individuals with DS, accounting for over 50% of hospitalizations and deaths. This increased susceptibility reflects a combination of anatomical, developmental, and immunological factors. Contributing conditions include midface hypoplasia, macroglossia, upper airway narrowing, airway malacia, hypotonia, swallowing dysfunction, gastroesophageal reflux, congenital heart disease, and immune dysregulation [3,4,5,6,7].

Among children with DS under three years of age, respiratory illnesses are the primary reason for hospital admission. The average hospital stay is two to three times longer than that of age-matched individuals without DS [8,9,10]. In particular, there is a higher incidence of respiratory tract infections, sleep-disordered breathing, and episodes of acute respiratory distress syndrome (ARDS), often triggered by recurrent viral infections, especially respiratory syncytial virus (RSV) [11,12].

Mortality rates due to respiratory conditions are markedly higher in both pediatric and adult populations with DS, with respiratory failure recognized as a major predictor of death in this population [13,14,15,16,17,18,19]. Furthermore, DS patients are also predisposed to pulmonary hypertension. In the literature has been described that overexpression of anti-angiogenic genes located on chromosome 21—such as COL4A3, endostatin, and RCAN1—is implicated in impaired vascular development and pulmonary hypoplasia, thereby increasing the risk of pulmonary hypertension in DS patients [20,21,22,23,24,25,26,27]. Structural lung abnormalities, including subpleural cysts, reduced alveolar numbers, and altered airway branching, have been observed in autopsy and imaging studies. These findings highlight the complex interplay of developmental, anatomical, and immunogenetic factors that shape the respiratory phenotype in DS [28,29] (Table 1).

Pulmonary sequestration (PS) represents a rare congenital pulmonary malformation that could theoretically compound respiratory complications in affected individuals. PS is defined as a non-functioning mass of lung tissue that has no identifiable communication with the normal bronchial tree and receives its blood supply from one or more anomalous systemic arteries [28,29,30,31,32]. Congenital pulmonary malformations have an estimated incidence ranging from 2.2% to 6.6% [33,34,35,36,37,38,39,40,41,42,43], although the true prevalence is likely lower, based on data from pediatric surgical series, post-mortem studies, and prenatal imaging, suggesting a range of 0.15–1.8% [44,45,46,47]. Among these, PS is the second most common anomaly. PS is classified into two types based on pleural covering: Intralobar sequestration (ILS) and extralobar sequestration (ELS) (Table 2).

ELS consists of a mass of pulmonary tissue completely separated from the normal lung by its own pleural covering, while ILS shares a pleural investment with adjacent lung tissue [48,49,50,51]. In approximately 80% of ELS cases, the arterial supply originates directly from the thoracic or abdominal aorta; around 15% are supplied by another systemic artery, and 5% by the pulmonary artery. Venous drainage is predominantly systemic—into the azygos, hemi-azygos, or inferior vena cava—although in about 25% of cases, partial or complete drainage into the pulmonary veins is observed. Most ELS lesions are located between the lower lobe of the lung and the diaphragm. Macroscopically, ELS appears as discrete, oval or pyramidal, gray-white to pink masses, ranging in size from 0.5 to 15 cm [32].

Most cases of ELS present within the first six months of life. Approximately one-quarter of affected infants develop symptoms shortly after birth, including respiratory distress or feeding difficulties [32]. Slightly older children typically present with respiratory symptoms and, occasionally, congestive cardiac failure [32].

ILS are more common than ELS. In contrast to ELS, no sex predilection has been identified, and ILS typically presents as an isolated anomaly [48,49,50,51]. ILS almost invariably involves the medial and posterior basal segments of the lower lobes, with the left hemithorax affected in approximately 60% of cases [32]. The arterial supply most often derives from the descending thoracic aorta (73%) and, less commonly, from the abdominal aorta (20%). Venous drainage occurs in more than 95% of cases directly into the pulmonary veins.

Although ILS may present at any age, unless detected during an antenatal ultrasound they rarely cause problems before the age of 2 years [32]. Presentation is usually the result of chronic or recurrent pneumonia or incidentally during imaging. A small number of patients present with high output cardiac failure, haemoptysis or massive intra-thoracic bleeding [32].

Pulmonary pseudosequestration, a variant of ILS involving partial communication with the bronchial tree, presents unique diagnostic and therapeutic challenges [32].

A high index of clinical suspicion should be maintained in any child presenting with recurrent chest infections, respiratory distress, or congestive heart failure without a clear cardiac etiology. In clinical practice, investigation typically begins with a chest radiograph. The radiologic appearance of PS is variable. Most ILS cases appear as well-defined, triangular masses oriented medially and posteriorly in the lung base. Air-fluid levels, indicative of communication with the bronchial tree, are observed in approximately 26% of cases. Additional radiographic findings include recurrent or persistent pneumonia. In rare cases, PS may manifest as a hyperlucent area or present with an air bronchogram [52,53]. Many ELS lesions, being small, may be radiographically occult [54]. Ultrasound is the recommended screening diagnostic examination for evaluating suspected supradiaphragmatic thoracic masses in children [52,53]. PS generally presents as a hyperechoic mass, and Doppler ultrasound may detect feeding arteries or draining veins, thus confirming the diagnosis [52,53].

Computed tomography (CT) is superior in delineating parenchymal abnormalities associated with PS [54,55,56,57,58,59,60,61,62,63]. The typical CT appearance is that of a complex mass, with or without cystic components. Occasionally, PS appears as a lesion composed of fluid- or air-filled microcysts, or as a large cavitary lesion with an air-fluid level. Magnetic resonance imaging (MRI), due to its multiplanar capability, allows detailed visualization of parenchymal lung changes as well as precise localization of systemic arterial supply and venous drainage [54,55,56,57,58,59,60,61,62,63]. In cases where non-invasive imaging is inconclusive, conventional angiography remains a valid diagnostic approach [34,60].

Recurrent infection represents the most common complication of ILS; however, the precise incidence and natural history of this condition remain unknown. While some authors advocate for surgical resection of all PS lesions due to diagnostic uncertainty and potential coexisting anomalies such as congenital pulmonary airway malformation (CPAM), others argue against surgery in asymptomatic neonates [44]. In managing asymptomatic lesions, particularly small ones, a risk-benefit analysis must be conducted, weighing surgical morbidity against the potential for future complications inherent to the sequestration itself.

Management of symptomatic PS typically involves surgical excision following resolution of infection [32,35]. Resection of ILS generally necessitates lobectomy, although segmental resection may be feasible in lesions identified prior to infection onset. In contrast, ELS lesions can often be managed with a sequestrectomy [32,42]. For both types of sequestration, precise identification and control of the aberrant vascular supply remain the critical components of surgical intervention. Although a broad and heterogeneous spectrum of respiratory involvement has been documented in individuals with DS, to date, no cases of pulmonary sequestration or pseudosequestration have been reported in this population [10,22].

Here we report the first known case of this association, highlighting its diagnostic complexity and clinical management within the broader framework of DS-related pulmonary vulnerability.

## 2. Case Description

**Figure 1:**
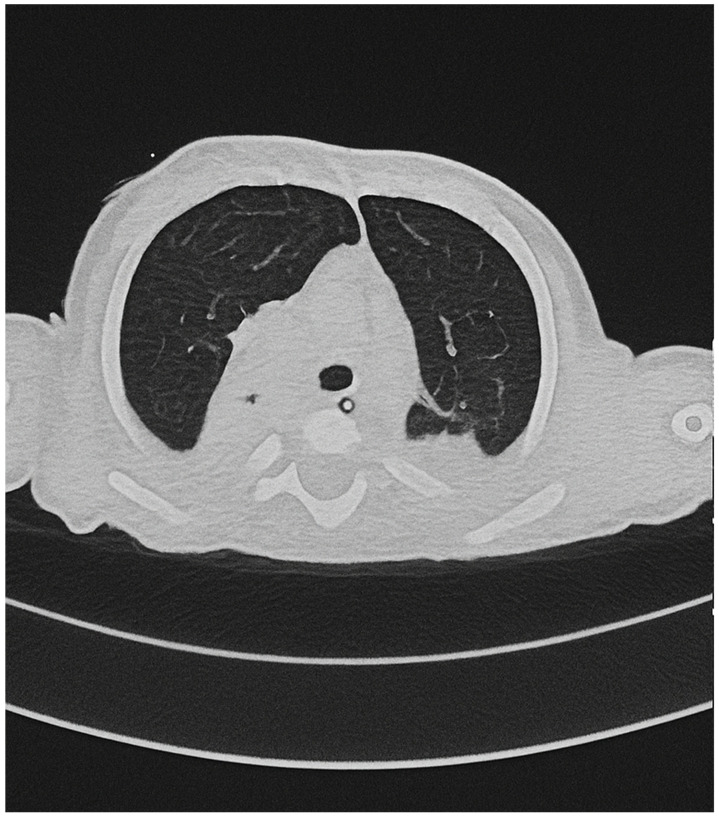
Contrast-enhanced CT angiography. The images demonstrate a right lower lobe lesion with systemic arterial supply from the celiac trunk, consistent with pulmonary pseudosequestration.

**Figure 2:**
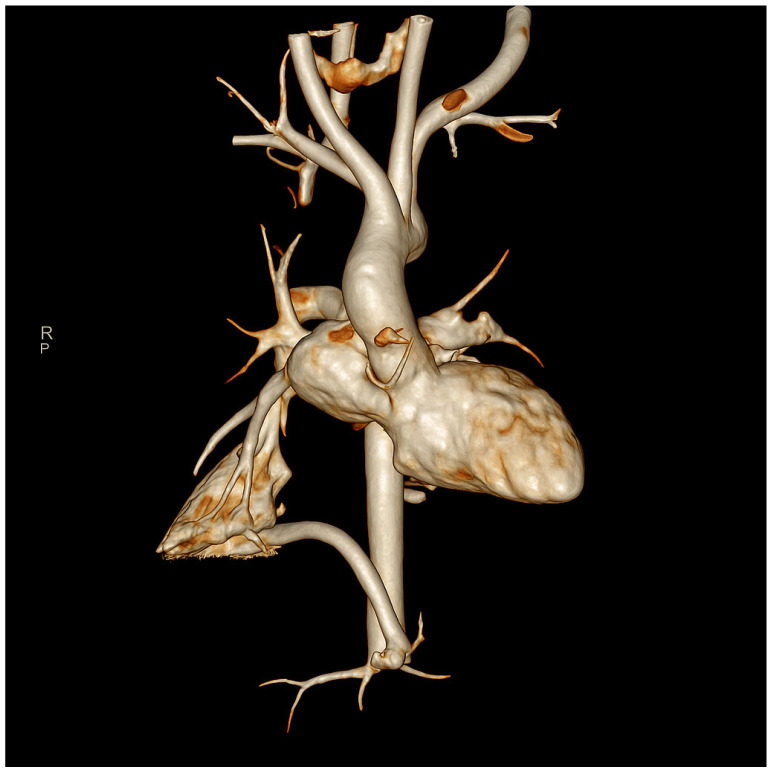
Three-dimensional volume-rendered reconstruction from contrast-enhanced CT angiography showing an anomalous systemic arterial supply to the right lower lobe arising from the celiac trunk, consistent with pulmonary pseudosequestration.

A. is a full-term female infant delivered by emergency cesarean section. Birth weight was 2.340 kg, consistent with small for gestational age. She was born to non-consanguineous parents. Prenatal ultrasound revealed fetal morphological abnormalities, prompting amniocentesis, which confirmed typical complete trisomy 21. No mosaicism or Robertsonian translocation was detected, and no further molecular analyses (FISH or microarray) were performed, as the karyotype was fully consistent with complete free trisomy 21. At birth, her APGAR scores were 6 at 1′ and 9 at 5′, and she presented with respiratory distress requiring CPAP support. In the first days of life, she was admitted to the Neonatal Intensive Care Unit with suspected early-onset sepsis and was treated with broad-spectrum antibiotics; blood cultures were negative. Hematologic follow-up was required in order to exclude transient myeloproliferative disorder associated with Down syndrome. At 21 days of life, she developed worsening respiratory distress associated with leukopenia and elevated C-reactive protein (66 mg/L),and was treated again for presumed sepsis with clinical improvement despite persistently negative cultures. At one month of age, she experienced a second episode of respiratory distress, without evidence of viral etiology. At four months of age, she developed acute respiratory failure with identification of RSV in nasal secretions, requiring admission to the Pediatric Intensive Care Unit. She required invasive mechanical ventilation, nitric oxide, and inotropic support for approximately 15 days, followed by weaning to high-flow nasal cannula (HFNC), which she continued for about one month before being progressively weaned off over the following two months. During this admission, in order to investigate the recurrent episodes of respiratory exacerbations, a contrast-enhanced chest CT demonstrated an intralobar pulmonary pseudosequestration in the right lower lobe. The anomalous systemic artery originated from the celiac trunk and drained via segmental branches of the inferior pulmonary vein, and the affected lung segment communicated with the bronchial tree (Figure 1 and Figure 2). At five months of age, she developed an Influenza A infection requiring HFNC support. Feeding difficulties were also significant. At one month of age, she developed diarrhea after switching to a standard formula and was therefore transitioned to a hydrolyzed formula. A subsequent attempt to reintroduce standard formula at two months again resulted in diarrhea. Beginning at four months of age, she required enteral nutrition via a nasogastric tube during her stay in sub-intensive unit. She underwent repeated nutritional and swallowing assessments and participated in a structured swallowing-training program, gradually transitioning from exclusive enteral feeding to full oral feeding over approximately 6–8 weeks. She ultimately tolerated her full daily intake without difficulty.

Given her complex condition, including atrial septal defect, severe obstructive sleep apnea, poor oral intake and hypotonia, a multidisciplinary team implemented a comprehensive program of nutritional rehabilitation and neuromotor therapy. As her feeding skills improved, she progressed from nasogastric tube feeding to exclusive oral intake and achieved satisfactory weight gain (4.550 kg at discharge). Surgical resection of the pulmonary sequestration is planned once she reaches a target weight of 6 kg.

## 3. Discussion

Pulmonary sequestration (PS) is a rare congenital anomaly characterized by the presence of non-functioning lung parenchyma that lacks normal communication with the tracheobronchial tree and receives its vascular supply from an aberrant systemic artery. Congenital pulmonary malformations have an estimated incidence ranging from 2.2% to 6.6% [20], rendering them relatively rare in comparison to acquired pulmonary diseases. It is the second most common congenital lung malformation after CPAM. PS is subdivided anatomically into intralobar sequestration (ILS)—which shares the same pleural covering as the adjacent lung—and extralobar sequestration (ELS)—which possesses its own pleural investment. A further variant, pseudosequestration, is defined by the presence of partial or anomalous communication with the bronchial tree, which makes diagnosis more complex and may lead to misclassification or delayed identification [32].

In the current case, a diagnosis of intralobar pulmonary pseudosequestration was made in a female infant with DS, representing the first documented association of these two conditions. This co-occurrence is of high interest, given the complex pulmonary phenotype and vascular dysregulation typical of trisomy 21. In Down syndrome, the overexpression of genes located on chromosome 21—particularly those involved in angiogenesis inhibition, such as COL4A3, endostatin (COL18A1), and RCAN1—is known to contribute to vascular immaturity, pulmonary hypoplasia, and increased risk for pulmonary hypertension. These anti-angiogenic signals may interfere with normal pulmonary vascular patterning and arborization, potentially promoting the persistence of aberrant systemic arteries and abnormal bronchopulmonary development, characteristic features of PS [20].

Based on these findings, we speculate a potential etiopathogenic link between DS and the development of PS. Pulmonary sequestration is thought to arise between the 4th and 8th weeks of gestation, originating from an accessory lung bud located caudal to the normal tracheobronchial tree, which subsequently acquires a systemic vascular supply.

The most widely accepted etiological hypothesis posits that sequestrations result from abnormal budding of the foregut or persistence of systemic arteries, leading to isolation of a lung segment from the main tracheobronchial tree. This accessory bud fails to connect with the developing airways and instead establishes anomalous connections with systemic arteries—most commonly the thoracic or abdominal aorta. In the case presented, the feeding vessel originated from the celiac trunk, a rare but described anatomical variant in ILS [40,44].

In the pediatric population, pulmonary sequestration—particularly the intralobar and pseudosequestration variants—can present with a highly variable clinical picture, ranging from asymptomatic incidental findings to severe respiratory compromise. While some cases are detected antenatally or during evaluations for unrelated conditions, others present with recurrent or persistent respiratory symptoms, often mimicking more common pediatric pulmonary disorders such as pneumonia or bronchiolitis [52,53].

It is therefore imperative for clinicians to maintain a high degree of diagnostic vigilance in any infant or child exhibiting recurrent lower respiratory tract infections, chronic cough, failure to thrive, or respiratory distress, especially when these symptoms are refractory to standard medical therapy or when no clear cardiac or infectious etiology is identified. In particular, peculiar radiographic findings or poor response to antibiotics should raise suspicion for an underlying congenital anomaly such as PS.

Pseudosequestration may share clinical features with ILS, including risks such as chronic aspiration, air trapping, and air-fluid level formation—all of which can predispose to secondary infection and recurrent inflammation [32]. When these malformations occur in syndromic patients, such as those with DS, even minor anomalies in ventilation or airway clearance can provoke disproportionately severe symptoms due to underlying hypotonia, immunodeficiency or airway malacia.

Some patients may also exhibit non-respiratory signs, including growth retardation, cyanotic episodes, feeding intolerance, or failure to gain weight, which may reflect chronic hypoxia or increased metabolic demand from repeated infections or subclinical respiratory compromise. In rare cases, congestive heart failure may develop, particularly when there is high-volume systemic arterial flow into the sequestered lung tissue, resulting in left-to-left shunting and volume overload [32,33,34].

Importantly, the absence of specific auscultatory findings or the presence of nonspecific crackles or diminished breath sounds may contribute to diagnostic delay. Therefore, any persistent abnormality on chest imaging, especially in the posterior basal segments of the lower lobes, warrants further investigation through advanced imaging modalities (e.g., CT angiography or MR angiography) to rule out congenital malformations such as PS or its variants.

Clinically, PS—especially pseudosequestration—presents a diagnostic challenge. While chest radiography may suggest a suspicious opacity or mass in the lower lobes, its sensitivity is limited, especially in cases of ELS or small lesions. Intralobar lesions, such as the one observed in our patient, typically appear as triangular masses oriented toward the mediastinum and located in the posterior basal segments of the lower lobes, with a predilection for the left hemithorax [54,55].

Moreover, lung specimens from DS infants frequently demonstrate subpleural cysts, reduced alveolar numbers, and simplified airway branching, which may further obscure the recognition of localized congenital anomalies on imaging or autopsy. These structural and vascular anomalies in individuals with DS increase their vulnerability to misdiagnosis and respiratory deterioration when pulmonary sequestration is present [64,65,66,67].

Ultrasound with Doppler is recommended as an initial imaging modality, particularly in infants, to assess for supradiaphragmatic masses and detect systemic arterial flow [53]. However, CT angiography remains the gold standard for accurately delineating anomalous vasculature and bronchial anatomy, thereby facilitating precise surgical planning [54].

In this context, it is important to outline the main differential diagnoses and clarify how CT angiography supported the diagnosis in our patient.

Intralobar pseudosequestration may resemble several congenital and acquired pulmonary conditions, making differential diagnosis essential, particularly when imaging is the main diagnostic tool. The most relevant differential diagnosis is congenital pulmonary airway malformation (CPAM), especially hybrid lesions with systemic arterial supply. However, CT angiography typically shows that CPAM retains a pulmonary arterial supply and often presents with multicystic parenchymal architecture, whereas pseudosequestration demonstrates exclusive systemic vascularization and a solid or mixed-density basal lesion with only aberrant or partial bronchial communication [32,54,57]. Another important differential consideration is bronchial atresia, which is characterized by a mucous-filled bronchocoele and distal hyperinflation but exhibits normal pulmonary vasculature, distinguishing it from the systemic arterial supply characteristic of pseudosequestration. Similarly, an aberrant systemic artery to normal lung (ASANL) may mimic sequestration but shows normal lung parenchyma without dysmorphic segments, with an isolated systemic vessel supplying otherwise typical tissue [68]. Bronchogenic cysts, although occasionally intrapulmonary, lack both anomalous arterial supply and parenchymal distortion presenting instead as well-circumscribed fluid-filled lesions [44]. Congenital lobar hyperinflation may also simulate an abnormal lower-lobe opacity; however, CT imaging demonstrates uniform hyperlucency with preserved pulmonary arterial perfusion. Finally, vascular malformations such as pulmonary arteriovenous malformation (PAVM) may be considered, but these lesions are characterized by a discrete nidus with dilated, directly connected feeding and draining vessels rather than a parenchymal malformation.

In our patient, the combination of exclusive systemic arterial supply from the celiac trunk, pulmonary venous drainage, dysmorphic intralobar basal parenchyma, and aberrant bronchial communication was diagnostic for intralobar pseudosequestration, effectively excluding these alternative entities [32,54,68].

In summary, while the clinical manifestations of pulmonary sequestration are different, the presence of recurrent localized infections, chronic respiratory symptoms, or failure to respond to standard treatments should prompt to consider this diagnosis. Early recognition is crucial to prevent complications such as irreversible lung damage, chronic infection, and cardiopulmonary deterioration, particularly in vulnerable populations such as children with trisomy 21, who are already predisposed to respiratory and immune dysfunction.

Management of PS is dictated by symptomatology, lesion size, and the presence of associated complications. Symptomatic lesions, particularly those causing respiratory distress, recurrent pneumonias, or cardiac overload, typically require surgical excision following clinical stabilization. In cases of ILS, the lesion is embedded within normal lung parenchyma, often necessitating lobectomy, although segmental resection may be feasible in early-detected, localized forms [39,60]. In contrast, sequestrectomy is generally preferred for extralobar sequestration (ELS), owing to the presence of a separate pleural covering [62].

A critical component of surgical planning is the identification and control of systemic arterial supply, which may originate from atypical sites such as the celiac, splenic, or subclavian arteries. Uncontrolled bleeding remains a significant intraoperative risk. In our patient, elective surgery was deferred to allow for weight gain and stabilization of associated comorbidities, including obstructive sleep apnea, hypotonia, and feeding difficulties—which are commonly observed in individuals with DS [62].

In recent years, endovascular embolization of the feeding artery has emerged as a minimally invasive alternative to surgery in selected cases, particularly in hemodynamically unstable neonates or when surgery must be delayed due to comorbidities. This technique involves the selective occlusion of the anomalous systemic vessel using coils, vascular plugs, or other embolic agents. Several case series and reports have demonstrated that embolization can effectively control symptoms such as hemoptysis or recurrent infections. In some cases, it may even eliminate the need for surgical resection, especially when a single feeding vessel is present and there is no communication with the bronchial tree [63,66,67].

Although promising, this approach is not without risk. Reported complications include post-embolization syndrome (fever, pain), pleural effusion, recanalization of the embolized artery, and persistent radiologic abnormalities requiring long-term monitoring [63,66,67]. Moreover, embolization precludes histopathologic evaluation of the lesion, which may be essential in cases where malignancy or infection is suspected.

Therefore, while surgical excision remains the gold standard, particularly in symptomatic or anatomically complex cases, endovascular embolization represents a valuable adjunct or bridge to surgery, especially in high-risk or fragile patients. In appropriately selected cases, it may serve as a definitive therapeutic strategy, provided that close clinical and radiological follow-up is ensured.

Mortality associated with PS is typically linked not to the lesion itself, but to coexistent congenital anomalies, such as congenital diaphragmatic hernia, total anomalous pulmonary venous return, or truncus arteriosus. In DS patients, who may already be burdened with cardiac anomalies (present in ~50% of cases), any added pulmonary lesion can significantly impact respiratory function and overall prognosis. Early identification and tailored intervention are thus crucial. Although long-term outcomes following surgical resection are generally favorable, complications such as chronic lung disease, asthma, or gastroesophageal reflux may persist, particularly in syndromic infants. Ongoing pulmonary follow-up and multidisciplinary care remain essential to optimize outcomes [68].

## 4. Conclusions

This case represents the first reported association between Down syndrome and pulmonary pseudosequestration, providing novel insights into the potential shared pathogenesis of vascular and pulmonary anomalies in DS. The complexity of the clinical presentation underscores the importance of integrated diagnostic approaches and individualized management strategies, particularly in vulnerable populations. The case also emphasizes the need for heightened awareness of rare congenital anomalies in children with trisomy 21 who present with respiratory distress, recurrent infections, or persistent imaging abnormalities.

Future studies may further clarify whether the genetic milieu of DS predisposes to aberrant pulmonary vascular development beyond currently recognized anomalies. Until then, each rare case such as this contributes a critical piece to the understanding of congenital pulmonary malformations in complex genetic syndromes.

## Figures and Tables

**Table 1:** Respiratory involvement in Down syndrome patients.

**Common**	
Pneumonias/recurrent respiratory infections	Immunologic screening Consider bronchoscopy to exclude bronchial malformations Exclude dysphagia
Sleep breathing disorders (e.g., obstructive sleep apnea syndrome, OSAS)	Night-time polygraphy or polysomnography in snoring, adeno/tonsillar hypertrophy, obesity, pulmonary hypertension
Laryngomalacia	Consider rhinofibrobronchoscopy In severe cases night-time polygraph or polysomnography Exclude dysphagia
Tracheobronchomalacia	Consider bronchoscopy in patients with noisy breathing, chronic cough, atypical or persistent wheezing
Tracheal bronchus	Consider in patients with recurrent right upper lobe pneumonia
Pulmonary hypertension	Consider in all patients with upper airway obstruction or unexplained hypoxia Echocardiography and cardiologic evaluation
Subpleural cysts	Incidental finding on CT scan Observation
Subglottic stenosis	Bronchoscopy
**Less Common**	
Post-obstructive pulmonary edema	Surgical evaluation
Altitude pulmonary edema	Persistent pulmonary hypertension of the newborn infant
Vascular ring	Bronchoscopy
Pulmonary hemorrhage	Abnormal RX, anemia or unexplained hypoxia Bronchoscopy
Interstitiopathies	Angio-TC

**Table 2:** Key features and management of Extralobar (ELS) vs. Intralobar (ILS) Pulmonary Sequestration.

Feature	Extralobar Sequestration (ELS)	Intralobar Sequestration (ILS)
Pleural covering	Own pleural lining, separate from adjacent lung parenchyma	Shares pleural lining with the involved pulmonary lobe
Age at presentation	≈25% present within first 6 months (respiratory distress, feeding difficulties)	Rarely symptomatic before age 2 unless detected prenatally; often discovered via recurrent pneumonia or incidentally
Arterial supply	80% from thoracic/abdominal aorta; 15% from other systemic arteries; 5% from pulmonary artery	≈73% from descending thoracic aorta; ≈20% from abdominal aorta
Venous drainage	Predominantly systemic (azygos, hemiazygos, IVC); ≈25% into pulmonary veins	>95% into pulmonary veins
Typical location	Between lower lobe and diaphragm	Medial and posterior segments of lower lobe (60% on left)
Clinical presentation	Neonatal respiratory distress, poor feeding; occasional high-output cardiac failure	Recurrent pneumonia, chronic cough, localized infections; occasional cardiac overload
Radiologic appearance	Well-defined mass; may be occult if small	Basal-medial triangular lesion; cavitation or hydro-air levels in ≈26%; CT shows microcysts/air–fluid levels
Diagnostic imaging	US/Doppler for supra-diaphragmatic masses; CT/MRI for vascular mapping and bronchial anatomy	CT angiography for anomalous vessels; MRI for multiplanar detail; conventional angiography if needed
Treatment	Surgical resection with early ligation of feeding vessel	Lobectomy or segmentectomy; antibiotics for infection; endovascular embolization as adjunct or temporizing measure

## Data Availability

All data generated or analyzed during this study are included in this published article.

## Abbreviations

The following abbreviations are used in this manuscript:

CHD	congenital heart defect
CPAM	congenital pulmonary airway malformation
CT	computed tomography
DS	Down syndrome
ELS	extralobar sequestration
ILS	intralobar sequestration
MRI	magnetic resonance imaging
PICU	pediatric intensive care unit
PS	pulmonary sequestration
RSV	respiratory syncytial virus
